# Emotional and Behavioral Changes and Related Factors of Firstborn School-Aged Compared to Same Age Only Children

**DOI:** 10.3389/fpubh.2022.822761

**Published:** 2022-03-03

**Authors:** Lulu Sheng, Bo Yang, Mary Story, Wenyi Wu, Xuan Xi, Yuanke Zhou, Yi Wen, Hong Wang, Qin Liu

**Affiliations:** ^1^School of Public Health and Management, Chongqing Medical University, Research Center for Medicine and Social Development, Innovation Center for Social Risk Governance in Health, Chongqing, China; ^2^Department of Family Medicine and Community Health and Duke Global Health Institute, Duke University, Durham, NC, United States; ^3^Chongqing Health Center for Women and Children, Chongqing, China

**Keywords:** firstborn children, only children, emotional, behavioral, related factors

## Abstract

**Objective:**

To compare the emotional and behavioral characteristics of firstborn children during the pregnancy of a second child and only children of school-age in urban districts of Chongqing, China, and to explore the influencing factors of emotional and behavioral problems.

**Methods:**

We recruited mothers of firstborn children and only children from two hospitals and one primary school using purposive sampling. Questionnaires and the Parental Child Behavior Checklist (CBCL) were used to collect basic information, family socioeconomic status, family atmosphere and emotional and behavioral characteristics of their children in the survey.

**Results:**

The sample consisted of 1,155 children, including 477 firstborn children and 678 only children. The average scores of internalizing (4.47), externalizing (6.05), total problems (22.04), and six emotional and behavioral of firstborn children were significantly lower than those of only children (*p* < 0.05). When adjusted for children's demographic, socioeconomic and family relationship covariates, the scores of firstborn children internalizing problems (β = −1.423, *p* = 0.000), externalizing problems (β = −0.661, *p* = 0.048), and total problems (β = −4.387, *p* = 0.000) were also significantly lower than those of only children. All children with more difficult parenting and development temperament, greater family economic pressure, poorer relationships between mother and child, less harmonious family atmosphere and father's permissive parenting style had more internalizing problems, externalizing problems and total problems (*p* < 0.05). Boys had more externalizing problems (β = 1.939, 95% CI = 1.380–2.497) and total problems (β = 4.908, 95% CI = 3.045–6.772) than girls.

**Conclusion:**

Firstborn children had fewer emotional and behavioral problems than their counterparts who were only children. This research helps to understand the social impact of the implementation of the two-child policy in multiple dimensions.

## Introduction

In order to better realize the harmonious development of population, economy, society, resources and environment, China's family planning policy has gone through several periods: the one-child policy implemented in 1980 through 2016, the selective two-child policy which started in 2013, and the universal two-child policy which began in 2016. After years of a national one-child policy, the arrival of the second child has changed the family structure of many only children.

In recent years, with the implementation of the universal two-child policy in China, the phenomenon of emotional and behavioral adjustment difficulties of the firstborn children in second-child families is commonly held belief, which has raised widespread societal concern in the society. However, there are relatively few studies focusing on firstborn children in China. In contrast, theoretical and empirical evidence has accumulated in foreign studies on families with two or more children, but the impact of the birth of a second child on the first child is still controversial (1–3). At the theoretical level, family crisis models and stressful life events models suggested that the emergence of a second child is a stressful life event that can bring psychological stress to family members and lead to negative psychological and behavioral changes (4, 5). According to ecological systems theory and the turning points theory, the birth of a second child may bring challenges to very young firstborn children, but it may also promote rapid development and growth, and not all children will experience significant changes (4). Previous research has focused on younger children or preschool age, and less on children of school age.

In addition, many domestic empirical studies have found that there may be differences in the mental health development between an only child and a child with siblings 6. However, these studies were not specific enough to determine the status of the non-only-child in the family, that is, it could not be determined that child with siblings is the firstborn child in a multi-child family. Moreover, these studies are relatively old, and family planning policies have changed a lot over time, which may limit the results of the studies. There is a lack of clarity about the differences between the first child and an only child in a two-child family since the implementation of the universal two-child policy in China. Therefore, the purpose of this study is to describe the emotional and behavioral characteristics and changes of firstborn children during the pregnancy of a second child in comparison to same age only children who are school age.

## Subjects and Methods

### Subjects

From March to December 2019, we recruited the mothers who went to the hospital to take the regular examination during the pregnant. Four hundred and seventy seven firstborn children who met the following inclusion criteria were recruited in obstetric clinics of two hospitals in urban Chongqing, including: (1) there is only one child in the family; (2) age of child is 6–12 years of age; (3) the mother was in the middle and third trimester of her second children, while 678 only children aged 6–12 years whose mother wasn't pregnant with a second child were recruited in a primary school in urban districts of Chongqing. The children whose parents divorced or from widowed families or children with mental disorders such as depression, autism were excluded.

The questionnaires were filled out by the mothers of the children. This study has been reviewed by the Ethics Committee of Chongqing Medical University, project title was “A prospective follow-up study on emotional and behavior changes and related mechanisms of firstborn children during role transition under the two-child policy.” Informed consent of the project was issued to the mothers of the children who met the inclusion criteria, and all the informed consents were taken as the objects of this investigation. A total of 1,282 paper questionnaires were handed out and filled out at hospitals and schools and 1,155 valid questionnaires (90.09%) were collected.

### Method

#### Survey Tools and Content

The questionnaire included the following contents: (1) basic information [e.g., children's age, gender, temperament type (easy to raise, difficult to raise, and slow development)], family type (nuclear family, extended family), primary carer of a child (parent, grandparent, other); (2) Family socioeconomic status: family annual income, family economic pressure, parents' education and occupation; Family atmosphere: parental relationship, parent-child relationship, family atmosphere, and parenting style (democratic, authoritarian, permissive and neglecting).

Based on clinical samples of children referred to child psychiatric services, Achenbach and colleagues developed an extensive checklist (6, 7), the Child Behavior Checklist for ages 6–18 (CBCL/6–18), which is a parent report form and assesses behavioral and emotional problems in children (4). The CBCL contains 113 items related to child functioning and problem behaviors in a variety of contexts. Parents indicated the degree or frequency of each behavior described in the item on a scale of 0 (not true), 1 (somewhat or sometimes true) or 2 (very true or often true). By summing 1s and 2s on all items, a total score on problem behavior was created.

From the 113 items, 8 narrowband syndromes were developed: Emotional Stability, Withdrawn, Somatic Complaints, Social Problems, Thought Problems, Attention Problems, Rule-breaking Behavior and Aggressive Behavior.

On the basis of a second order factor analysis, three broadband syndromes were formed. (1) Emotional Stability, Withdrawn and Somatic Complaints were combined to form a composite score on Internalizing. (2) Rule-breaking Behavior and Aggressive Behavior formed a composite score on Externalizing. (3) The 113 items formed a composite score on total problems. The higher the score, the more serious the problem.

In this study, the internal consistency reliability test and correlation analysis were conducted for the CBCL scales filled out by 1,155 mothers, which were respectively, expressed by Cronbach α coefficient and Spearman correlation coefficient. The internal consistency Cronbach α coefficient of the total CBCL table for children was 0.918, the correlation coefficients between the syndromes and the overall scale ranged from 0.633 to 0.869, and the correlation coefficients between internalizing and externalizing problems and the total scale were 0.863 and 0.911, respectively. All correlation coefficients were significant at the level of 0.001.

### Quality Control

First, all investigators were familiar with the investigation environment and process through the pilot study, conducted in the hospital and problems were discussed and solved during the study. Second, before the formal study began, the investigators had been trained uniformly to ensure that they had a good understanding of the project background, research methods and content. Third, the questionnaires filled out by the mother was collected in clinics and schools, and the investigators checked whether the questionnaire information was missing and whether there were logical errors. Fourth, Epidata 3.1 was used for double entry of the collected questionnaires and error correction was checked to ensure the accuracy of the data.

### Statistical Treatment

IBM SPSS Statistics 21.0 was used for data analysis. Categorical variables were described by frequency and percentage, and continuous variables were described by mean ± standard deviation. Chi-square test was used to compare the differences in the basic conditions of the children, and rank sum test was used to compare the differences in emotional and behavioral scores of the children. Using multiple linear regression, the scores of internalizing problems, externalizing problems, and total problems of the firstborn children and only children were taken as the dependent variables, demographic characteristics, socio-economic status of the family, family relationship, and parenting style were taken as the independent variables, to explore the influencing factors of the children's emotional and behavioral problems. In all analyses *p* < 0.05 was considered statistically significant.

## Results

### Information of the Target Children

Among the 1,155 children surveyed, 568 (49.2%) were boys and 585 (50.6%) were girls. The mean age of the firstborn children was lower than that of only children (8.30 ± 1.75 vs. 9.28 ± 1.80, *p* < 0.05). The differences of family type, persons responsible for fostering and education, type of temperament, family economic pressure, parents' education level, occupation, parents' relation, family atmosphere, and parenting style between firstborn children and only children were statistically significant (*p* < 0.05), while other characteristics of the two groups were similar, see Table 1.

**Table 1:** Comparison of children characteristics, socio-economic characteristics, family relationship between firstborn and only children, *n* (%), Chi-square test.

**Factors**	**All children**	**Firstborn children**	**Only children**	***χ** ^**2**^*	***P***
	**(*n =* 1,155)**	**(*n =* 477)**	**(*n =* 678)**		
**Children characteristics**					
Age of children				50.502^**^	0.000
6–9 years	795 (69.01)	382 (80.59)	413 (60.91)		
10–12 years	357 (30.99)	92 (19.41)	265 (39.09)		
Children gender				0.174	0.676
Male	568 (49.18)	231 (48.53)	337 (49.78)		
Female	585 (50.65)	245 (51.47)	340 (50.22)		
Family type				15.683^**^	0.000
Nuclear family	370 (32.03)	121 (25.64)	249 (36.73)		
Extended family	780 (67.53)	351 (74.36)	429 (63.27)		
Primary carer of a child				6.547^*^	0.038
Parenting	911 (78.87)	364 (76.47)	547 (80.68)		
Parenting and grand-parenting	216 (18.70)	95 (19.96)	121 (17.85)		
Grand-parenting	27 (2.34)	17 (3.57)	10 (1.47)		
Type of temperament				14.579^**^	0.001
Easy to raise	960 (83.12)	421 (88.82)	539 (80.45)		
Difficult to raise	89 (7.71)	27 (5.70)	62 (9.25)		
Slow development	95 (8.23)	26 (5.49)	69 (10.30)		
**Socio-economic situation of the family**					
Total annual household income (RMB)				106.289^**^	0.000
<120,000	581 (50.30)	161 (35.15)	420 (63.44)		
120,001–240,000	379 (32.81)	186 (40.61)	193 (29.15)		
240,001–360,000	89 (7.71)	60 (13.10)	29 (4.38)		
>360,000	71 (6.15)	51 (11.14)	20 (3.02)		
Family financial pressure				34.82^**^	0.000
No pressure	48 (4.16)	14 (2.95)	34 (5.07)		
Less pressure	196 (16.97)	83 (17.51)	113 (16.84)		
Medium pressure	574 (49.70)	273 (57.59)	301 (44.86)		
Higher pressure	255 (22.08)	93 (19.62)	162 (24.14)		
Great pressure	72 (6.23)	11 (2.32)	61 (9.09)		
Father education				78.834^**^	0.000
Junior high school and below	158 (13.68)	37 (7.77)	121 (17.87)		
Senior high school	298 (25.80)	87 (18.28)	211 (31.17)		
College and undergraduate	625 (54.11)	300 (63.03)	325 (48.01)		
Master degree or above	72 (6.23)	52 (10.92)	20 (2.95)		
Mother education				62.591^**^	0.000
Junior high school and below	137 (11.86)	31 (6.51)	106 (15.63)		
Senior high school	326 (28.23)	99 (20.80)	227 (33.48)		
College and undergraduate	647 (56.02)	317 (66.60)	330 (48.67)		
Master degree or above	44 (3.81)	29 (6.09)	15 (2.21)		
Father's occupation				114.723^**^	0.000
Leadership and management	93 (8.05)	44 (9.30)	49 (7.27)		
Professional and technical staff	281 (24.33)	155 (32.77)	126 (18.69)		
Office clerks	103 (8.92)	59 (12.47)	44 (6.53)		
Business service personnel	248 (21.47)	59 (12.47)	189 (28.04)		
Workers	178 (15.41)	33 (6.98)	145 (21.51)		
Personal entrepreneurship	202 (17.49)	105 (22.20)	97 (14.39)		
Other	42 (3.63)	18 (3.80)	24 (3.56)		
Mother's occupation				76.271^**^	0.000
Leadership and management	52 (4.50)	23 (4.88)	29 (4.30)		
Professional and technical staff	266 (23.03)	141 (29.94)	125 (18.52)		
Office clerks	157 (13.59)	76 (16.14)	81 (12.00)		
Business service personnel	249 (21.56)	59 (12.53)	190 (28.15)		
Workers	74 (6.41)	18 (3.82)	56 (8.30)		
Personal entrepreneurship	137 (11.86)	64 (13.59)	73 (10.81)		
Full-time mothers	142 (12.29)	51 (10.83)	91 (13.48)		
Other	69 (5.97)	39 (8.28)	30 (4.44)		
**Family relationship and parenting style**					
Parental relationship				16.707^**^	0.000
Very good	615 (53.25)	268 (56.42)	347 (51.18)		
Relatively good	376 (32.55)	164 (34.53)	212 (31.27)		
General	162 (14.03)	43 (9.05)	119 (17.55)		
Father-child relationship				5.163	0.076
Very good	693 (60.00)	295 (62.11)	398 (58.79)		
Relatively good	358 (31.00)	149 (31.37)	209 (30.87)		
General	101 (8.74)	31 (6.53)	70 (10.34)		
Mother-child relationship				3.334	0.189
Very good	836 (72.38)	358 (75.37)	478 (70.50)		
Relatively good	280 (24.24)	103 (21.68)	177 (26.11)		
General	37 (3.20)	14 (2.95)	23 (3.39)		
Family atmosphere				12.886^**^	0.002
Very harmony	275 (23.81)	126 (26.47)	149 (21.98)		
Comparative harmony	742 (64.24)	312 (65.55)	430 (63.42)		
General	137 (11.86)	38 (7.98)	99 (14.60)		
Father's parenting style				13.586^**^	0.004
Democratic	715 (61.90)	321 (68.59)	394 (58.54)		
Authoritarian	211 (18.27)	79 (16.88)	132 (19.61)		
Permissive	112 (9.70)	37 (7.91)	75 (11.14)		
Neglecting	103 (8.92)	31 (6.62)	72 (10.70)		
Mother's parenting style				12.499^**^	0.006
Democratic	845 (73.16)	369 (78.85)	476 (70.94)		
Authoritarian	196 (16.97)	60 (12.82)	136 (20.27)		
Permissive	76 (6.58)	28 (5.98)	48 (7.15)		
Neglecting	22 (1.90)	11 (2.35)	11 (1.64)		

* *p < 0.05;*

** *p < 0.01*.

### Comparison of Scores of Emotional and Behavioral Problems in Firstborn and Only Children

The rank sum test results of 477 firstborn children and 678 only children showed that the scores of emotional and behavioral syndromes and comprehensive problems of firstborn children were significantly lower than those of only children, except for thought problems and rule-breaking behavior (*p* < 0.05), as shown in Table 2.

**Table 2:** Comparison of emotional and behavioral scores between firstborn children and only children, Mean (SD), Rank sum test.

**Dependent variables**	**Total score of problems**	**All children** **(*n =* 1,155)**	**Firstborn and only children**			
			**Firstborn children**	**Only children**	***Z***	***P***
			**(*n =* 477)**	**(*n =* 678)**		
**Narrowband syndromes**						
Emotional stability	26.00	2.52 (2.56)	2.05 (2.32)	2.84 (2.68)	−5.873	0.000
Withdrawn	16.00	1.83 (1.98)	1.40 (1.66)	2.13 (2.14)	−6.254	0.000
Somatic complaints	22.00	1.39 (1.75)	1.01 (1.45)	1.66 (1.90)	−6.611	0.000
Social problems	22.00	3.10 (2.54)	2.79 (2.34)	3.33 (2.65)	−3.333	0.001
Thought problems	30.00	1.76 (1.97)	1.59 (1.76)	1.88 (2.10)	−1.578	0.115
Attention problems	20.00	5.05 (3.57)	4.15 (3.23)	5.69 (3.66)	−7.271	0.000
Rule-breaking behavior	34.00	2.21 (1.91)	2.12 (1.93)	2.28 (1.90)	−1.588	0.112
Aggressive behavior	36.00	4.48 (3.78)	3.92 (3.54)	4.86 (3.89)	−4.513	0.000
**Comprehensive problems**						
Internalizing	64.00	5.74 (5.24)	4.47 (4.41)	6.63 (5.58)	−7.146	0.000
Externalizing	70.00	6.69 (5.28)	6.05 (5.05)	7.14 (5.40)	−3.669	0.000
Total	206.00	25.67 (17.91)	22.04 (16.03)	28.23 (18.71)	−5.579	0.000

### Factors Influencing Emotional and Behavioral Problems of Firstborn Children and Only Children

#### Simple Linear Regression Analysis

The results showed that the scores of internalizing, externalizing, and total problems of firstborn children were significantly lower than those of only children (*p* < 0.01), and children with difficult parenting and slow development temperament, greater family financial pressure, lower parental education level, poor parental relationship, poor father-child relationship, poor mother-child relationship, not very harmonious family atmosphere, non-democratic father's or mother's parenting style scored higher on internalizing, externalizing and total problems (*p* < 0.05). The scores of externalizing and total problems were higher in boys (*p* < 0.01), and the older children, the higher their internalizing problems scores (*p* < 0.05), see Table 3.

**Table 3:** Simple and multiple linear regression on emotional and behavior problems of all children surveyed.

**Variable**	**Model 1** ^**b**^						**Model 2** ^**c**^					
	**Internalizing problems**		**Externalizing problems**		**Total problems**		**Internalizing problems**		**Externalizing problems**		**Total problems**	
	**β**	**95% CI of B**	**β**	**95% CI of B**	**β**	**95% CI of B**	**β**	**95% CI of B**	**β**	**95% CI of B**	**β**	**95% CI of B**
Type of children												
Firstborn	−2.160^**^	−2.761, −1.558	−1.093^**^	−1.710, −0.477	−6.186^**^	−8.256, −4.116	−1.659^**^	−2.284, −1.035	−0.661^*^	−1.315, 0.007	−4.189^**^	−6.214, −2.164
Only children^a^												
Age of children	0.169^*^	0.005, 0.334	−0.102	−0.268, 0.064	−0.089	−0.651, 0.474	−0.009	−0.171, 0.154	-	-	-	-
Children gender												
Male	0.523	−0.081, 1.128	2.089^**^	1.491, 2.687	5.760^**^	3.719, 7.802	-	-	1.939^**^	1.380, 2.497	4.908^**^	3.045, 6.772
Female^a^												
Type of temperament												
Difficult to raise	4.398^**^	3.308, 5.487	4.148^**^	3.033, 5.263	17.005^**^	13.290, 20.721	3.085^**^	2.004, 4.166	2.336^**^	1.255, 3.417	10.937^**^	7.332, 14.542
Slow development	3.968^**^	2.911, 5.025	2.613^**^	1.530, 3.695	11.960^**^	8.354, 15.566	2.819^**^	1.778, 3.860	1.303^*^	0.267, 2.340	7.422^**^	3.965, 10.878
Easy to raise^a^												
Age of mother	0.057	−0.018, 0.131	−0.045	−0.120, 0.030	−0.061	−0.317, 0.194	-	-	-	-	-	-
Family type												
Nuclear family	0.014	−0.635, 0.662	−0.431	−1.084, 0.223	−1.035	−3.251, 1.181	-	-	-	-	-	-
Extended family^a^												
The upbringing and education of children												
Parenting and grand parenting	0.45	−1.615, 2.215	0.902	−1.180, 2.984	2.864	−4.195, 9.924	-	-	-	-	-	-
Parenting	0.016	−1.957, 1.988	0.525	−1.464, 2.514	1.485	−5.258, 8.228	-	-	-	-	-	-
Grand parenting^a^												
Family financial pressure												
Medium pressure	1.121^**^	0.354.1.887	1.099^**^	0.327, 1.871	4.141^**^	1.528, 6.754	0.797^*^	0.068, 1.527	0.581	−0.140, 1.302	2.556^*^	0.153, 4.960
Higher pressure	2.216^**^	1.365, 3.066	2.372^**^	1.515, 3.228	8.436^**^	5.536, 11.336	0.944^*^	0.113, 1.774	1.077^*^	0.253, 1.902	3.469^*^	0.718, 6.221
Less pressure^a^												
Father education												
College and undergraduate	1.891^**^	0.636, 3.147	1.503^*^	0.234, 2.773	6.622^**^	2.333, 10.910	0.74	−0.607, 2.087	0.427	−0.914, 1.768	2.636	−1.836, 7.109
Senior high school	2.109^**^	0.783, 3.435	1.472^*^	0.131, 2.813	7.166^**^	2.635, 11.697	0.303	−1.198, 1.805	0.116	−1.377, 1.609	1.548	−3.432, 6.528
Junior high school and below	3.034^**^	1.596, 4.473	2.445^**^	0.991, 3.900	10.934^**^	6.020, 15.848	0.666	−1.018, 2.349	0.696	−0.979, 2.371	3.962	−1.624, 9.548
Master degree or above^a^												
Mother education												
College and undergraduate	1.803^*^	0.227, 3.380	1.539	−0.053, 3.132	6.197^*^	0.808, 11.587	0.873	−0.811, 2.556	0.771	−0.904, 2.446	2.367	−3.221, 7.955
Senior high school	2.600^**^	0.974, 4.226	2.347^**^	0.704, 3.989	9.493^**^	3.934, 15.052	1.191	−0.619, 3.001	1.065	−0.735, 2.866	3.501	−2.505, 9.508
Junior high school and below	2.771^**^	1.014, 4.528	1.394	−0.380, 3.169	7.195^*^	1.188, 13.201	1.043	−0.953, 3.040	−0.267	−2.247, 1.713	−0.486	−7.092, 6.119
Master degree or above^a^												
Parental relationship												
Relatively good	1.737^**^	1.075, 2.399	1.312^**^	0.643, 1.981	5.361^**^	3.097, 7.624	0.391	−0.341, 1.123	−0.281	−1.009, 0.446	−0.214	−2.640, 2.212
General	2.095^**^	1.202, 2.989	2.394^**^	1.491, 3.297	8.034^**^	4.980, 11.089	−0.643	−1.725, 0.439	−0.589	−1.665, 0.486	−3.007	−6.595, 0.582
Very good^a^												
Father-child relationship												
Relatively good	1.756^**^	1.099, 2.412	2.026^**^	1.366, 2.686	7.199^**^	4.969, 9.429	0.359	−0.426, 1.144	0.753	−0.028, 1.533	2.544	−0.060, 5.147
General	2.791^**^	1.717, 3.866	2.829^**^	1.749, 3.909	10.531^**^	6.880, 14.182	0.908	−0.355, 2.172	0.439	−0.819, 1.696	2.709	−1.486, 6.904
Very good^a^												
Mother-child relationship												
Relatively good	2.448^**^	1.757, 3.138	2.501^**^	1.808, 3.194	8.871^**^	6.523, 11.220	0.956^*^	0.150, 1.763	1.300^**^	0.500, 2.099	3.673^**^	1.006, 6.340
General	4.261^**^	2.581, 5.941	5.107^**^	3.420, 6.793	16.243^**^	10.527, 21.959	2.030^*^	0.274, 3.787	3.323^**^	1.581, 5.066	8.561^**^	2.749, 14.373
Very good^a^												
Family atmosphere												
Comparative harmony	2.205^**^	1.500, 2.910	2.505^**^	1.798, 3.211	8.958^**^	6.575, 11.342	1.219^**^	0.511, 1.926	1.401^**^	0.698, 2.103	4.907^**^	2.564, 7.250
General	4.097^**^	3.052, 5.143	4.471^**^	3.423, 5.519	16.427^**^	12.893, 19.960	2.006^**^	0.810, 3.202	2.327^**^	1.138, 3.516	8.624^**^	4.658, 12.590
Very harmonious^a^												
Father's parenting style												
Authoritarian	1.480^**^	0.686, 2.273	1.257^**^	0.465, 2.048	5.671^**^	2.992, 8.351	0.478	−0.309, 1.266	0.338	−0.446, 1.122	1.94	−0.674, 4.554
Permissive	1.955^**^	0.926, 2.985	3.127^**^	2.100, 4.154	9.883^**^	6.404, 13.361	1.007^*^	−0.002, 2.015	2.511^**^	1.519, 3.503	6.881^**^	3.573, 10.190
Neglecting	2.134^**^	1.066, 3.202	2.887^**^	1.821, 3.952	10.279^**^	6.671, 13.887	−0.006	−1.172, 1.160	1.101	−0.059, 2.262	2.946	−0.925, 6.817
Democratic^a^												
Mother's parenting style												
Authoritarian	1.898^**^	1.096, 2.701	2.135^**^	1.325, 2.945	7.730^**^	4.997, 10.463	0.515	−0.291, 1.322	0.692	−0.111, 1.495	2.341	−0.337, 5.020
Permissive	1.695^**^	0.481, 2.909	1.621^**^	0.397, 2.846	7.396^**^	3.264, 11.528	0.238	−0.938, 1.415	0.207	−0.962, 1.376	1.784	−2.114, 5.682
Neglecting	3.488^**^	1.298, 5.678	2.092	−0.118, 4.301	9.953^**^	2.497, 17.409	2.041	−0.098, 4.180	−0.592	−2.724, 1.539	1.643	−5.467, 8.753
Democratic^a^												

a *Reference group*.

b *Model 1 is simple linear regression on emotional and behavior problems of object children*.

c *Model 2 is multiple linear regression on emotional and behavior problems of all children, adjusted the factors with statistical differences in model 1 (Internalizing problems adjusted factors: type and age of children, type of temperament, family financial pressure, father and mother education, parental, father-child and mother-child relationship, family atmosphere, parenting style*.

*Externalizing and total problems adjusted factors: type and gender of children, type of temperament, family financial pressure, father and mother education, parental, father-child, and mother-child relationship, family atmosphere, parenting style). No data is available because the corresponding variables are not included in the analysis*.

* *p < 0.05;*

** *p < 0.01*.

#### Multiple Linear Regression Analysis

The multiple linear regression model also showed that the scores of internalizing, externalizing, and total problems of firstborn children were significantly lower than those of only children (*p* < 0.05), and children with difficult parenting and slower development temperament, greater family economic pressure, mother-child relationship issues, not very harmonious family atmosphere, and father's permissive parenting style had higher scores in internalizing, externalizing, and total problems (*p* < 0.05). In addition, boys had higher scores of externalizing (β = 1.939, 95% CI = 1.380, 2.497) and total problems (β = 4.908, 95% CI = 3.045, 6.772), see Table 3.

## Discussion

Our findings indicate that firstborn children had fewer emotional and behavioral problems than their counterparts who were only children. This study has played a good role in supporting China's family planning, helping mothers to reduce their worries about having a second child. This study examined the emotional and behavioral characteristics and influencing factors of firstborn children 6–12 years of age, whose mothers were in the second and third trimesters of their pregnancy. In addition to directly assessing the emotional and behavioral characteristics of firstborn children, this study also compares the differences between firstborn children and same age only children.

This study adapted a scientific rigorous scale, the CBCL scale for emotional and behavioral problems instead of a self-made scale, which addresses limitations of previous studies and helps better understand emotional and behavioral characteristics of the first child during his mother's pregnancy with a second child. Gottlieb and Baillies (8) found that firstborn children have less separation reaction and dependent behaviors than only children, and Harris et al. (9) found that firstborn children during the mother's pregnancy show more clinging behaviors, sleep disturbances and tantrums than expected. Other studies using the CBCL scale to examine the emotional and behavioral characteristics of school-age children generally conduct comparative analysis on the birth order of children, lacking the comparison between two groups.

In this study, both univariate and multivariate analysis results showed that firstborn children had fewer emotional and behavioral problems than only children of the same age. By comparing the basic characteristics and combining the influencing factors of the emotional and behavioral characteristics of the two types of children, the results show that the two types of children have large differences in these characteristics, and these differences are important influencing factors for emotional and behavioral problems.

First, the results show that the scores of internalizing, externalizing and total problems of children whose mothers report they are easier to raise are lower than only child. The proportion of firstborn children who mothers report are easier to raise is higher, while the proportion of firstborn children with difficulty in raising is lower. The children's temperament survey in this study adopted the “three temperament” theory proposed by Thomas and Chess (10). Consistent with the results of other studies using this theory (11), this study found that children who were easier to raise showed fewer emotional and behavioral problems than other children. Previous studies (12) have shown that children who mothers perceive are easier to raise are less susceptible to the influence of the environment, thus showing less emotional and behavioral problems, while children with perceived difficulty in raising are on the contrary. Easily raised children have shown positive and regular reactions since early infancy, and can quickly adapt to new environments, have more positive emotions, and have mild emotional reactions. Such children are less affected by the environment (12, 13), while children with difficulty in raising are more likely to be affected by the environment, thus showing higher rates of disorders and behavioral problems (12, 14).

Second, the results show that children with strong mother-child relationship and a harmonious family atmosphere score lower on internalizing, externalizing, and total problems. In this study, the proportion of firstborn children with a perceived strong mother-child relationship and a harmonious family atmosphere is higher than that of families with only children. According to the ecological systems theory and family-systems theory, the interaction and connection between family environment and family systems will have a significant impact on the social and emotional development of children (15, 16). The reason can be explained as follows: According to the social learning theory, children mostly observe their parents (17). They may copy the emotions and expressions of their parents, as well as emotion regulation strategies, because parents are the main attachment objects of the children (18). Mothers are the primary caregivers of young children. Children can influence their emotional regulation methods by observing mothers' warm emotional expressions (19) and then show positive emotional and behavioral characteristics. However, a stressful family environment is more likely to lead to children's mental health problems (4), and the influence of the family atmosphere on the eldest son may be related to the Emotional Security Hypothesis (20). The hypothesis suggests that discordant family relationships may impair children's emotional security and the extent to which they feel safe in the home environment, and that emotional insecurity in turn has a negative impact on children's behavior and social interaction. Further, positive and harmonious family relationships help to reduce the potential harmful influence of family on children, so that they show less emotional and behavioral problems (21).

Third, the results show that children with father's permissive parenting style score higher in internalizing, externalizing and total problems. In this study, there are more only children families with father's permissive parenting style than firstborn children. In univariate analysis, parental parenting style was related to children's emotional and behavioral problems, but after adjusting for other factors, only father's permissive parenting style was still related to children's emotional and behavioral problems. For firstborn children, it may be due to the influence of the mother's pregnancy that the mother's own energy and physical conditions have changed, and the mother's attention and companionship to firstborn children have decreased. During this period, the father's parenting style and companionship to firstborn children play a more important role. For only children, studies have shown that (22) there is a greater correlation between the quality of father's companionship and children's psychological and behavioral adaptation. Therefore, even after adjusting other factors, the permissive parenting style of the father is still the main influencing factor for children's emotional and behavioral problems. Different parenting styles can directly affect children's emotional and behavioral expression and make unique and independent contributions to the development of children's social behavior. Fathers' parenting styles can significantly predict children's social behaviors (23), in the parenting of children, father as models play the role of guiding children to the outside world (24). Studies have shown that (25) fathers' doting and indulging behaviors of their children may lead to more emotional and behavioral problems in children.

Fourthly, the results show that children with high family economic pressure score higher in internalizing, externalizing and total problems. In this study, families with firstborn children had less economic pressure than families with only children. In this study, family economic stress was reported by mothers. In terms of mothers' emotions, studies found that (26–29) emotional problems such as high levels of stress in mothers may affect the adaptation and development of children's emotions and behaviors. Many previous studies have shown that greater economic pressure is the main reason why women of child-bearing age are reluctant to have a second child, which is consistent with the results of this study. We speculate that parents with low family economic pressure have enough economic ability to have a second child and pay attention to the quality of offspring cultivation, pay attention to the emotional and behavioral problems of children and give positive guidance to them. However, parents whose families are under great economic pressure worry that their energy is not enough to guarantee the quality two childrens' growth.

This study also found that boys showed more externalizing problems and total problems, which may be related to the level of activities of both boys and girls, as well as social and cultural factors. From infancy, boys have stronger mobility than girls (30) so girls are more likely to internalize the difficulties and stress they encounter. Meanwhile, the “Problem Suppression-Facilitation Model” proposed by Weisz et al. (31) indicates that some cultural factors inhibit the development of children's specific problems while promoting the development of other problems. Chinese social and cultural education methods and expectations for boys and girls are quite different. Girls are often required to be more introverted and quiet (32), while boys are expected to show more extroverted and active behaviors, resulting in more parental constraints on girls' extroverted behaviors than boys, thus showing that girls have fewer externalizing problems than boys (33).

## Limitations and Future Directions

The study has some limitations. First, the firstborn children and only children were recruited from two hospitals and a primary school in Chongqing urban districts, respectively, which may cause selection bias. The differences in basic characteristics of the two groups of children may influence their emotional and behavioral problem characteristics, so it is recommended that in future research, firstborn children and only children from different sources should be recruited. In addition, the questionnaire was based on mother's self-report of their child's behavior and not direct observation, mother's appraisal of children may be influenced by many factors, for example, expectant mother's emotion may interplay her appraisal to her child. Therefore, if conditions permit, the method of combining maternal questionnaires with direct observation of the emotional and behavioral performance of children would make the data collected more robust. Moreover, we used a cross-sectional design to examine the emotional and behavioral characteristics of firstborn children during their mother's second or third trimester and there was no follow up period to assess emotional and behavioral changes after the birth of the second child. Prospective cohort follow-up is needed to determine the relationship and changes between role changes and emotional and behavioral problems of firstborn children, as well as causal associations between various influencing factors and emotional and behavioral problems of firstborn children.

## Conclusions

In conclusion, school-aged firstborn children's emotional and behavioral problems are relatively fewer than their counterpart only children in our study. These results need to be confirmed in further studies conducted in more regions with bigger sample size. In future intervention studies, we may pay more attention to the externalizing problems of boys and children with difficulty in raising. Adopt effective ways to adjust the relationship between mothers and children, and provide scientific guidance for fathers to bring up their children to effectively prevent the occurrence of emotional and behavioral problems or reduce the severity of the problems.

## Data Availability Statement

The original contributions presented in the study are included in the article/Supplementary Material, further inquiries can be directed to the corresponding author/s.

## Ethics Statement

The studies involving human participants were reviewed and approved by the Ethics Committee of Chongqing Medical University. Written informed consent to participate in this study was provided by the participants' legal guardian/next of kin.

## Author Contributions

MS, HW, and QL were involved in conception and design, data collection, data interpretation, and critical review for this article. BY was involved in data analysis for this article. LS and QL were involved in manuscript drafting. WW, XX, YZ, and YW were involved in data collection, case diagnosis, and confirmation for this article. All authors reviewed the manuscript. All authors contributed to the article and approved the submitted version.

## Funding

This project was supported by Scientific and Technological Research Program of Chongqing Municipal Education Commission (Grant No. KJQN201800425).

## Conflict of Interest

The authors declare that the research was conducted in the absence of any commercial or financial relationships that could be construed as a potential conflict of interest.

## Publisher's Note

All claims expressed in this article are solely those of the authors and do not necessarily represent those of their affiliated organizations, or those of the publisher, the editors and the reviewers. Any product that may be evaluated in this article, or claim that may be made by its manufacturer, is not guaranteed or endorsed by the publisher.

## References

[1] Oh W Volling BL Gonzalez R. Trajectories of children's social interactions with their infant sibling in the first year: a multidimensional approach. J Fam Psychol. (2015) 29:119–29. 10.1037/fam0000051 25664367 PMC4410802

[2] Song JH Volling BL. Coparenting and children's temperament predict firstborns' cooperation in the care of an infant sibling. J Fam Psychol. (2015) 29:130–5. 10.1037/fam0000052 25581467 PMC4324056

[3] Kendrick C Dunn J. Sibling quarrels and maternal responses. Dev Psychobiol. (1983) 19:62–70. 10.1037/0012-1649.19.1.62

[4] Volling BL Brenda L. Family transitions following the birth of a sibling: an empirical review of changes in the firstborn's adjustment. Psychol Bull. (2012) 138:497–528. 10.1037/a0026921 22289107 PMC3341504

[5] Chen BB Wang Y Liang J Tong L. And baby makes four: biological and psychological changes and influential factors of firstborn's adjustment to transition to siblinghood. Adv Psychol Sci. (2016) 24:863–73. 10.3724/SP.J.1042.2016.00863

[6] Achenbach TM Rescorla L. Manual for the ASEBA School-Age Forms and Profiles. Burlington: University of Vermont, Research Center for Children, Youth, and Families (2001).

[7] Petot D Rescorla L Petot JM. Agreement between parent-and self-reports of Algerian adolescents' behavioral and emotional problems. J Adolesc. (2011) 34:977–86. 10.1016/j.adolescence.2010.11.011 21163517

[8] Gottlieb LN Baillies J. Firstborns' behaviors during a mother's second pregnancy. Nurs Res. (1995) 44:356–62. 10.1097/00006199-199511000-00007 7501490

[9] Harris MJ John K Sharp R. The effects of a mother's second pregnancy on the firstborn child. Aust N Z J Obstet Gyn. (1989) 29:319–21. 10.1111/j.1479-828X.1989.tb01753.x 2619680

[10] Bidjerano T. Thomas and chess classification of infant. In: Goldstein S. Naglieri J. A. editors. Encyclopedia of Child Behavior and Development. Boston, MA: Springer (2011). 10.1007/978-0-387-79061-9_2912

[11] Zhong Y. An Analysis of temperament characteristics of preschoolers in Nantong city and its influencing factors. Matern Child Health Care China. (2009) 24:36–9.

[12] Eisenberg N Valiente C Spinrad TL Cumberland A Liew J Reiser M. Longitudinal relations of children's effortful control, impulsivity, and negative emotionality to their externalizing, internalizing, and co-occurring behavior problems. Dev Psychol. (2009) 45:988–1008. 10.1037/a0016213 19586175 PMC2775424

[13] Feng JJ. Research on Attachment, Temperamental Characteristics and Maternal Attachment of Children Aged 2–3. [master's thesis]. Shanghai, IL: Shaanxi Normal University (2008).

[14] Liu Q Zhang Q Huang X. Study on temperament-based structural equation model for behavioral problems of preschool children. Matern Child Health Care China. (2013) 28:436–8.

[15] Cox MJ Paley B. Understanding families as systems. Curr Dir Psychol Sci. (2003) 12:193–6. 10.1111/1467-8721.01259

[16] Morris AS Silk JS Steinberg L Myers SS Robinson LR. The role of the family context in the development of emotion regulation. Soc Dev. (2007) 16:361–88. 10.1111/j.1467-9507.2007.00389.x 19756175 PMC2743505

[17] Liu C Wu XC Chen LL Xing XW. The impact of family of origin on co-parenting. J S China Norm U. (2013) 6:74–80.

[18] Su LP Kubricht B Miller R. The influence of father involvement in adolescents' overall development in Taiwan. J Adolesc. (2017) 59:35–44. 10.1016/j.adolescence.2017.05.010 28554076

[19] Denham SA. Dealing with feelings: how children negotiate the worlds of emotions and social relationships. Behaviour. (2007) 1:1–48.

[20] Kolak AM Volling BL. Coparenting moderates the association between firstborn children's temperament and problem behavior across the transition to siblinghood. J Fam Psychol. (2013) 27:355–64. 10.1037/a0032864 23750518 PMC3698979

[21] Kolak AM Vernon-Feagans L. Family-level coparenting processes and child gender as moderators of family stress and toddler adjustment. Infant Child Dev. (2010) 17:617–38. 10.1002/icd.577 19907670 PMC2774923

[22] Deng LY Wang XT Xiong YY Li YT Li BL. The relationship among paternal accompany, maternal emotion and psychosocial adjustment of primary school firstborn children in two-children families. Chin J Clin Psychol. (2020) 28:254–60.

[23] Russell A Hart CH Robinson CC Olsen S. Children's sociable and aggressive behaviour with peers: a comparison of the US and Australia, and contributions of temperament and parenting styles. Int J Behav Dev. (2003) 27:74–86. 10.1080/01650250244000038

[24] Zhang YQ Liu L Sun DL Yin X Chen Z Wu CC. Relationship between parenting styles and conduct problems in 3-year-old preschool children. Chin J Nerv Ment Dis. (2017) 43:229–33.

[25] Ye TT Chen Y Fan F. Relationship between parenting Style and conduct problems in 3-year-old pre-schoolers. In: Compilation of the 9th Annual Academic Conference of Chinese Sleep Research Association. (2016). p. 1.

[26] Eamon MK Zuehl RM. Maternal depression and physical punishment as mediators of the effect of poverty on socioemotional problems of children in single-mother families. Am J Orthopsychiat. (2010) 71:218–26. 10.1037/0002-9432.71.2.218 11347362

[27] Guajardo NR Snyder G Petersen R. Relationships among parenting practices, parental stress, child behavior, and children's social-cognitive development. Infant Child Dev. (2009) 18:37–60. 10.1002/icd.578

[28] Mcclure EB Brennan PA Hammen C Brocque R. Parental anxiety disorders, child anxiety disorders, and the perceived parent-child relationship in an australian high-risk sample. J Abnorm Child Psych. (2001) 29:1–10. 10.1023/A:1005260311313 11316331

[29] Xing XP Li MM Yin TT. Relations between maternal negative emotion and preschooler's externalizing problem behavior: the moderating effects of home chaos. J Psychol Sci. (2018) 41:842–8.

[30] Xu J Chen TN Ding XL Shi W Bao YL. Preliminary application of Achenbach Child Behavior Checklist. Chin J Woman Child Health Res. (2015) 26:194–6.

[31] Weisz JR Somsong SMA Chaiyasit W Bahr WMS Achenbach TM Walter B. Epidemiology of behavioral and emotional problems among Thai and American Children: parent reports for ages 6 to 11. J Am Acad Child Psy. (1987) 26:890–7. 10.1097/00004583-198726060-00014 3429409

[32] Yang RM. The Study of Effects of Perinatal Factors on Preschoolers' Psychological Behavior. master's thesis. Nanjing, IL: Nanjing Medical University (2011).

[33] Tao GT Qiu JH Li BL Zeng WX Xu J. Comparison of behavior development between only children and children with siblings: six-year follow-up study. Chin Ment Health J. (1996) 10:1–5.

