# Supplementary material for: Emotional and Behavioral Changes and Related Factors of Firstborn School-Aged Compared to Same Age Only Children
Source: Front Public Health. 2022 Mar 3;10:822761. doi: 10.3389/fpubh.2022.822761 (PMC8929573; doi:10.3389/fpubh.2022.822761)
Supplement: Supplementary file 1 [file Data_Sheet_1.PDF]

Supplementary Material

1 Supplementary Table

Supplementary table 1 Reliability test and correlation analysis of CBCL

	Emotional and behavioral syndromes								Comprehensive problems		
	Emotional Stability	Withdrawn	Somatic Complaints	Social Problems	Thought Problems	Attention Problems	Rule-breaking Behavior	Aggressive Behavior	Internalizing problems	Externalizing problems	Total problems
α reliability coefficient											
Firstborn children	0.733	0.689	0.670	0.716	0.617	0.819	0.676	0.822	0.727	0.808	0.907
Only children	0.755	0.746	0.694	0.736	0.658	0.826	0.601	0.838	0.767	0.824	0.912
Whole children	0.752	0.739	0.695	0.732	0.639	0.830	0.637	0.833	0.765	0.817	0.918
Spearman α correlation coefficient											
Internalizing problems	0.891	0.820	0.756	-	-	-	-	-	-	-	-
Externalizing problems	-	-	-	-	-	-	0.856	0.965	-	-	-
Total problems	0.785	0.683	0.633	0.822	0.729	0.844	0.772	0.869	0.863	0.911	-

Note:[a]Spearman P values were 0.000 in the correlation analysis.

- No data is available because the corresponding variables are not included in the anal
